# Novel gas producing *Vibrio cholerae*: a case report of gastroenteritis with acute kidney injury

**DOI:** 10.1099/acmi.0.000005

**Published:** 2019-04-17

**Authors:** Jeevan Malaiyan, Anandan Balakrishnan, Sowmya Nasimuddin, Kamalraj Mohan, PradeepRaj Meenakshi-Sundaram, Selvam Mamandur-Devarajan, Sumathi Gnanadesikan, Mohanakrishnan Kandasamy, Nithyalakshmi Jayakumar, Dhevahi Elumalai, Gokul G. Ra

**Affiliations:** 1 Department of Microbiology, Sri Muthukumaran Medical College Hospital and Research Institute, Affiliated to The Tamil Nadu Dr. M.G.R. Medical University, Chikkarayapuram, Chennai 600 069, India; 2 Department of Genetics, Dr. ALM Post Graduate Institute of Basic Medical Sciences, University of Madras, Taramani campus, Chennai 600113, India; 3 Department of General Medicine, Sri Muthukumaran Medical College Hospital and Research Institute, Affiliated to The Tamil Nadu Dr. M.G.R. Medical University, Chikkarayapuram, Chennai 600 069, India

**Keywords:** diarrhea, gas production, *Vibrio cholerae*, sequencing

## Abstract

**Background:**

Bacterial characterization is important in clinical and epidemiological studies. We herein report the first case of gas-producing *Vibrio cholera* gastroenteritis with acute kidney injury.

**Case presentation:**

A 30-year-old female presented to the emergency department with complaints of about ten episodes of watery diarrhea, four episodes of vomiting and elevated serum urea/creatinine levels. Although the bacteria were first misidentified as *Vibrio furnissii* by gas production on carbohydrate fermentation and triple sugar iron agar, it was later confirmed as *Vibrio cholerae* by 16 S rRNA gene sequencing and specific PCR. The treatment regimen was followed as for *Vibrio* species with intravenous fluids, ciprofloxacin and doxycycline. The patient recovered without relapse.

**Conclusions:**

Literature survey from the PubMed database shows no gas-producing *Vibrio cholerae* isolate being reported in the world. Further, genotype studies are warranted to look into the gas production of *Vibrio cholerae *.

## Introduction

The genus *Vibrio* is a member of the family *Vibrionaceae* and consists of 37 recognized species. *Vibrio* species are ubiquitously distributed in marine and estuarine environments worldwide. Only twelve species are potential human pathogens causing diarrhea, septicemia and extra intestinal infections, e.g. wound infections [1, 2]. *Vibrio* species are facultative anaerobes, motile by a single polar flagellum, and are oxidase positive (except *Vibrio metschnikovii and Vibrio proteus*) and do not produce gas from glucose (except *Vibrio furnissii *) [2, 3]. *Vibrio cholerae* *e* causes the diarrheal disease cholera, by the ingestion of contaminated water and food [4]. According to the World Health Organization (WHO) report, 3.5 million people get infected and 21 000–143 000 deaths occur worldwide each year [5–7]. Cholera cases and deaths in African and South Asian countries have accounted for 99% of the total cholera cases worldwide [6]. Localized epidemics are also frequent; half a million cases have been attributed to the current outbreak in Yemen, Lusaka and Zambia [8, 9]. Over 200 serogroups of *V. cholerae* have been recognized so far, and the most common serogroups are O1 and O139, which cause epidemic cholera [6]. Although many phenotype and serogroups have been identified, we herein report for the first time a case of gas-producing *V. cholerae* strain causing gastroenteritis with acute kidney injury.

## Case report

A 30-year-old female presented to the emergency department with complaints of about ten episodes of severe watery diarrhea during the past 1 day. She also had complaints of four episodes of vomiting. There was no history of abdominal pain. She had consumed fried sea food a day before the onset of diarrhea and vomiting. On examination, the patient’s vitals were normal with pulse rate 76/bpm, blood pressure 110/70 mmHg and respiratory rate 18/bpm. Signs of dehydration were observed. No significant abnormalities were detected in cardiovascular, respiratory and central nervous systems. On palpation abdomen was soft and non tender with no organomegaly and no evidence of fluid.

Routine blood and urine investigations along with blood culture, urine culture, stool culture and hanging drop were done. Total white blood cell (WBC) count was increased with the value of 15 400 cells/cumm with 80% of neutrophils. Serum electrolytes were within normal limits. Renal function test shows Urea 64 (reference value 15–40 mg dl^−1^) and Creatinine 4.2 (reference value 0.5–1.4 mg dl^−1^) was elevated. The blood and urine cultures were found to be negative. On macroscopic examination the stool sample was found to be watery and non purulent. On microscopic examination by hanging drop actively motile (darting motility) bacteria were observed. The clinicians were immediately notified with the report and the patient was started with intravenous fluids, Injection Metronidazole 500 mg QID for 7 days and Ciprofloxacin 500 mg bd for 7 days. The patient condition improved symptomatically with renal parameters also returning within the normal range within 3 days after treatment.

In microbiological laboratory, the sample was inoculated in alkaline peptone water and was plated on to blood agar, MacConkey agar and thioglycollate bile salt sucrose (TCBS) agar, and was incubated for 24 h. The colonies on blood agar were flat with hemodigestion. On MacConkey agar, pale non lactose fermenting (NLF) colonies were observed. On TCBS agar, they were yellow in colour confirming sucrose fermentation. With the growth positive on TCBS agar and pale NLF colonies, the presence of *Vibrio* species is confirmed, which was further substantiated by the following biochemical tests. It was oxidase positive, catalase negative, produced gas in glucose fermentation and triple sugar iron agar with acid/acid with no H2S, citrate positive, urease negative, indole positive, nitrate reduction positive, lysine and ornithine positive, sucrose and mannitol utilization positive, Voges–Proskauer test positive and resistant to 50 U polymyxin B.

The antibiogramwas done by Kirby–Bauer disc diffusion method with standard antibiotic discs from HIMEDIA, India following Clinical & Laboratory Standards Institute (CLSI) guidelines (M100-S15, 2012) showed sensitivity to gentamicin, ceftriaxone, ciprofloxacin, doxycycline and resistance to amikacin, ampicillin and nalidixic acid.

**Fig. 1.**
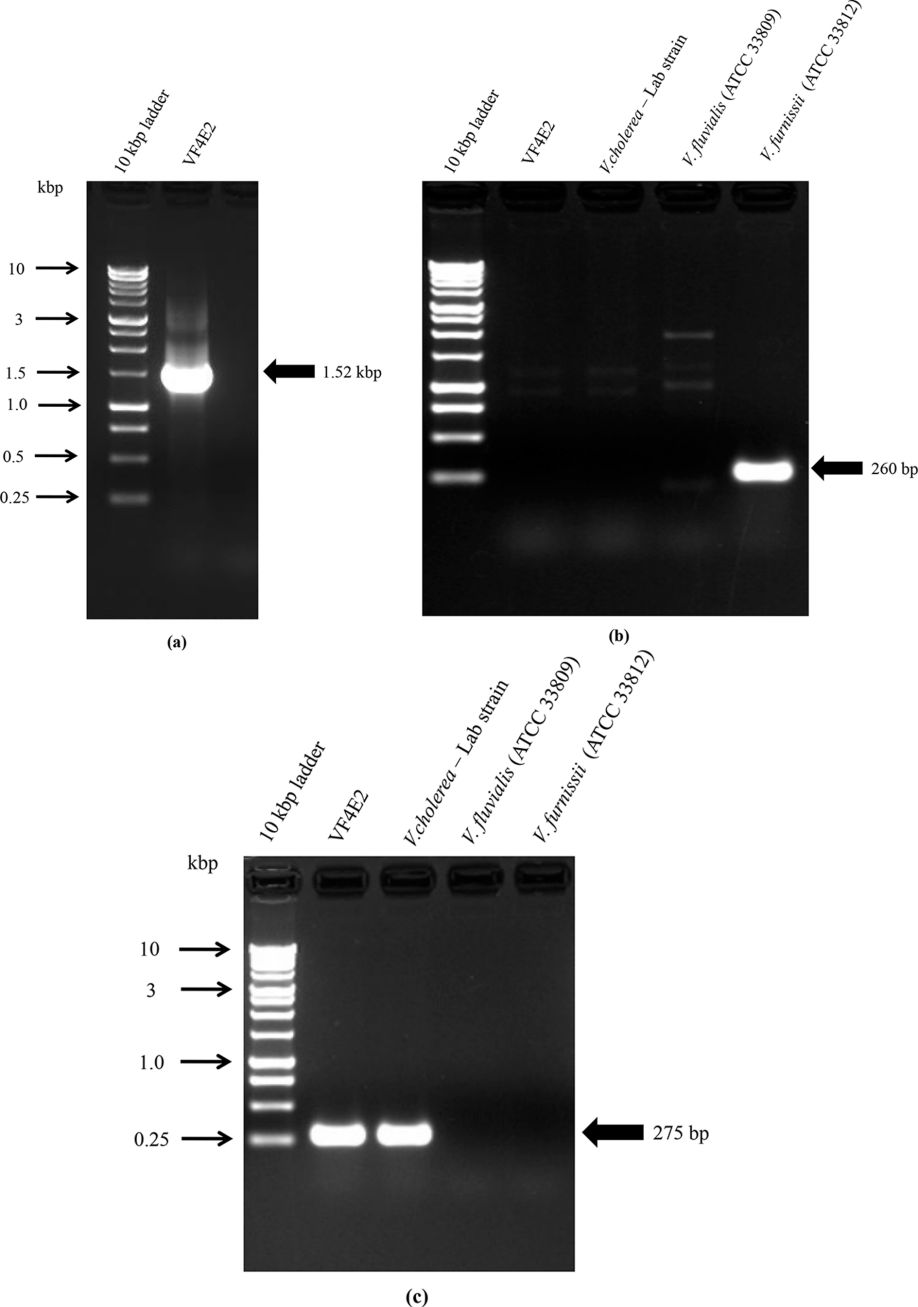
(a) Genus *Vibrio *16S rRNA PCR (1.52 kbp), VF4E2 – clinical isolate. (b) Specific PCR for *V. furnissii* (260 bp), VF4E2 – clinical isolate. Negative control: *V. cholerae* – lab strain and *V. fluvialis* (ATCC 33809). Positive control: *V. furnissii* (ATCC 33812). (c) Specific PCR for *V. cholera* (275 bp), VF4E2 – clinical isolate. Positive control: *V. cholerae* – lab strain. Negative control: *V. fluvialis* (ATCC 33809) and *V. furnissii* (ATCC 33812).

Based on growth on selective medium TCBS and strong gas production evidence in carbohydrate fermentation and triple sugar iron agar, the clinical isolate (VF4E2) was suspected as *V. furnissii *. PCR and Sanger dideoxy sequencing were carried out. Amplification and sequencing the 16S rRNA gene (1.52 kb) was performed, with PrimeSTAR GXL DNA polymerase and primers based on the method described by Takajo *et al.* [10]. Sequence of the PCR product of 16S rRNA gene of the strain (accession no. MH885569) was compared with the sequences in the DDBJ/EMBL/GenBank and EzBioCloud databases on 25 August 2018. The result indicated that *V. cholerae* showed the highest homology (99%) with our isolate. The specific PCR for *V. furnissii* and *V. cholerae* was performed according to the method of Schirmeister *et al*. and Neogi *et al*., respectively [Table 1], and was found to be negative for *V. furnissii* and positive for *V. cholerae* [11, 12, Fig. 1]. Serology of *V. cholerae* was determined by slide agglutination test with polyvalent O1, and monovalent Ogawa, and Inaba antisera. Considering these findings, the clinical isolate (VF4E2) was identified as gas-producing *V. cholerae* serogroup O1, biovar El Tor, serotype Ogawa strain.

**Table 1.** Target gene and PCR primers used for species identification.

Target	Primer	Sequence	Amplicon size	Reference
16S rRNA gene	1F	AGAGTTTGATCMTGGCTCAG	1.52 kbp	[10]
16S rRNA gene	1517R	TACGGTTACCTTGTTACGAC		
*toxR* *V. furnissii *	Vfurn-toxR2-fo	AGACGCTGATCTCGATCCAC	260 bp	[11]
*toxR* *V. furnissii *	Vfurn-toxR2-re	TTGTCAAAGACCGCCAGAC		
*toxR* *V. cholerae *	VC toxR 403F	GAAGCTGCTCATGACATC	275 bp	[12]
*toxR* *V. cholerae *	VC toxR 678R	AAGATCAGGGTGGTTATTC		

## Discussion

Cholera, caused by the Gram-negative bacterium *V. cholerae *, can be treated using antibiotics along with administration of intravenous fluids and oral rehydration salts [7]. *V. cholerae* isolates from primary culture are identified by colonial appearance, Gram stain, biochemical testing, serology (agglutination with specific antisera), 16S rRNA PCR, specific PCR and sequencing [2, 10–12]. Epidemic strains of *V. cholerae* O1 can be differentiated into El Tor and classical biotypes and further subdivided into Inaba, Ogawa and Hikojima serotypes. Strains not belonging to serogroup O1 are generally referred to as *V. cholerae* non O1 [2]. *V. cholerae* carries several virulence-related genes to provoke pathogenic processes in the infected hosts. The key virulence factors of serogroups O1 and O139 include cholera toxin (CT), which is responsible for profuse watery diarrhea [7].

A case of gastroenteritis was documented in India and identified as *V. furnissii* based on acid/acid with gas on triple sugar iron agar with no H_2_S and matrix-assisted laser desorption/ionization time-of-flight mass spectrometry [13]. Whereas in the present case, the bacteria were first misidentified as *V. furnissii* by gas production on carbohydrate fermentation and triple sugar iron agar, it was later confirmed as *V. cholerae* by 16S rRNA gene sequencing and specific PCR, suggesting that genetic characterization is mandatory along with phenotype identification, when rare species are isolated. Cases of severe, acute renal failure during severe attacks of diarrhea caused by *V. cholerae* have been described in Israel and United States [14, 15]. In our case, severe diarrhea lead to acute kidney injury with elevated urea/creatinine levels and the patient recovered without relapse by treatment with intensive infusions of fluids, electrolytes, sodium bicarbonate and antibiotics. By the twenty-first century, more than enough information about the pathogen, identification, treatment, control strategies, its epidemiology and genetics was revealed. However, there is still fear of cholera cases and outbreaks in developing countries [6]. We are herewith presenting the first strain of gas-producing *V. cholera* *e* and a literature survey from the PubMed database shows no such isolate being reported in the world. To summarize, a 30-year-old female with gastroenteritis was first misidentified as *V. furnissii* by gas production, it was later confirmed as *V. cholerae* by targeted PCR and sequencing. Further, genotype studies are warranted to look into the gas production of *V. cholerae *.
